# Supplementary figures and images for: Metabolomics of a cell line-derived xenograft model reveals circulating metabolic signatures for malignant mesothelioma
Source: PeerJ. 2022 Jan 4;10:e12568. doi: 10.7717/peerj.12568 (PMC8740518; doi:10.7717/peerj.12568)

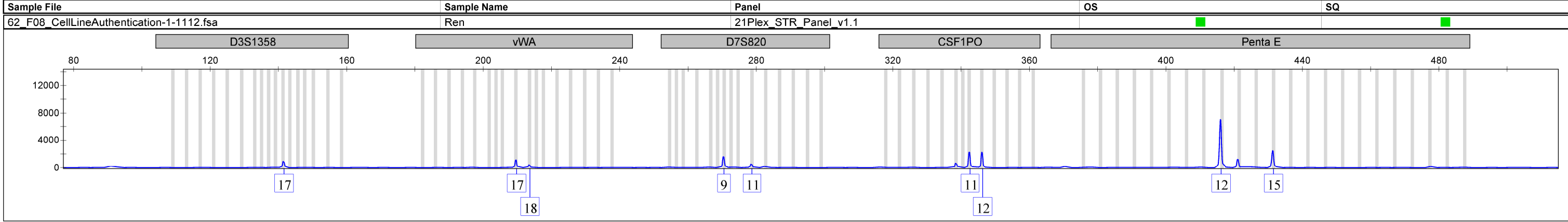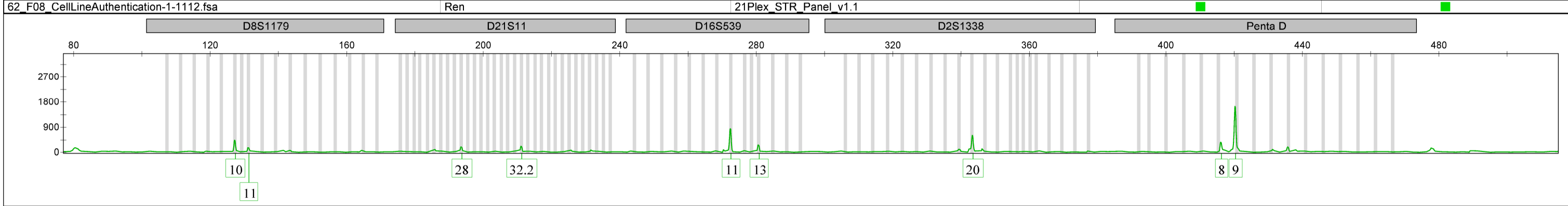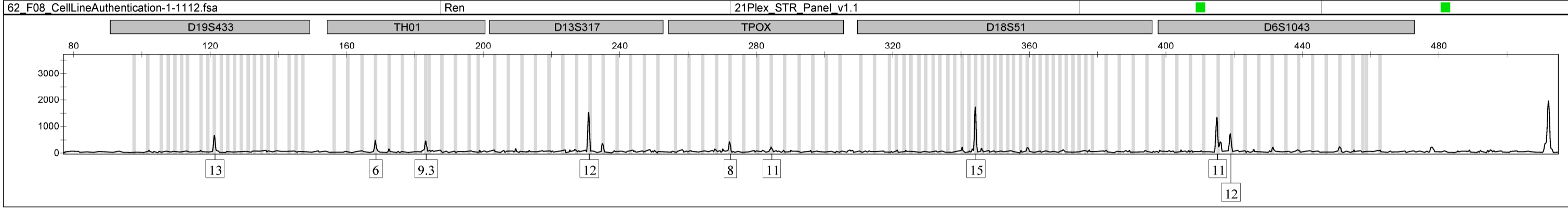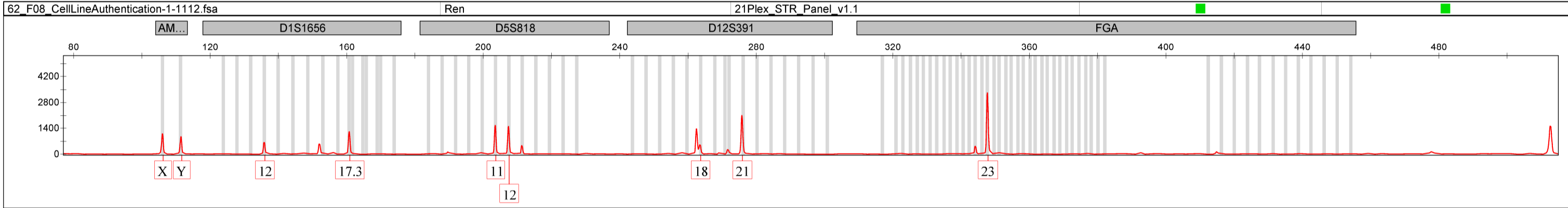

Supplement: Supplemental Information 2 [file peerj-10-12568-s002.pdf]

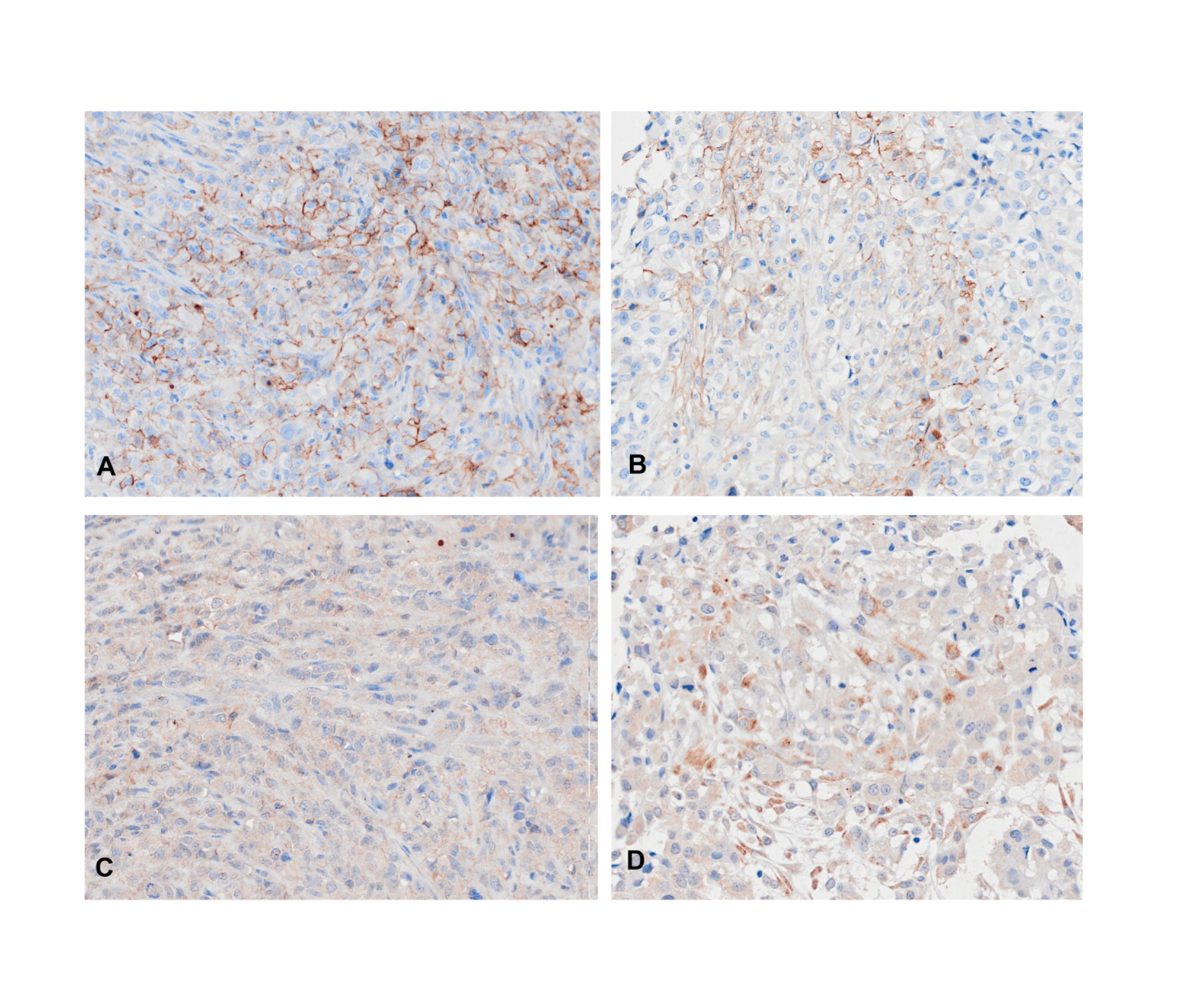

Supplement: Supplemental Information 6 — Immunohistochemical stains of SLC1A5 in MM CDX model (A) and MM patient (C); of SLC7A5 in MM CDX model (B) and MM patient (D). [file peerj-10-12568-s006.jpg]
