# Supplementary material for: Metabolomics of a cell line-derived xenograft model reveals circulating metabolic signatures for malignant mesothelioma
Source: PeerJ. 2022 Jan 4;10:e12568. doi: 10.7717/peerj.12568 (PMC8740518; doi:10.7717/peerj.12568)
Supplement: Supplemental Information 4 — FC: fold change; adj.P-Value: adjusted P-Value. [file peerj-10-12568-s004.docx]

| Gene | logFC | P-Value | adj.P-Value |
| --- | --- | --- | --- |
| *SLC1A3* | 0.66045806 | 1.70E-07 | 1.55E-06 |
| *SLC1A5* | -0.1577949 | 0.01555082 | 0.04091824 |
| *SLC7A5* | 0.81092973 | 4.24E-08 | 4.44E-07 |

**Table S1**: Differential SLC gene analyses.

FC: fold change; adj.P-Value: adjusted P-Value.
